# Comparative analysis of the chloroplast genomes of *Rosa* species and RNA editing analysis

**DOI:** 10.1186/s12870-023-04338-0

**Published:** 2023-06-14

**Authors:** Chengwen Gao, Teng Li, Xia Zhao, Chuanhong Wu, Qian Zhang, Xiangzhong Zhao, Mingxuan Wu, Yihong Lian, Zhiqiang Li

**Affiliations:** 1 Medical Research Center, The Affiliated Hospital of Qingdao University, Qingdao University, Qingdao, 266000 Shangdong China; 2 School of Public Health, Qingdao University, Qingdao, 266000 Shangdong China

**Keywords:** *Rosa*, Chloroplast genome, RNA editing, RNA-Seq, Phylogenetic analysis

## Abstract

**Background:**

The genus *Rosa* (Rosaceae) contains approximately 200 species, most of which have high ecological and economic values. Chloroplast genome sequences are important for studying species differentiation, phylogeny, and RNA editing.

**Results:**

In this study, the chloroplast genomes of three *Rosa* species, *Rosa hybrida*, *Rosa acicularis*, and *Rosa rubiginosa*, were assembled and compared with other reported *Rosa* chloroplast genomes. To investigate the RNA editing sites in *R. hybrida* (commercial rose cultivar), we mapped RNA-sequencing data to the chloroplast genome and analyzed their post-transcriptional features. *Rosa* chloroplast genomes presented a quadripartite structure and had highly conserved gene order and gene content. We identified four mutation hotspots (*ycf3-trnS, trnT-trnL, psbE-petL*, and *ycf1*) as candidate molecular markers for differentiation in the *Rosa* species. Additionally, 22 chloroplast genomic fragments with a total length of 6,192 bp and > 90% sequence similarity with their counterparts were identified in the mitochondrial genome, representing 3.96% of the chloroplast genome. Phylogenetic analysis including all sections and all subgenera revealed that the earliest divergence in the chloroplast phylogeny roughly distinguished species of sections *Pimpinellifoliae* and *Rosa* and subgenera *Hulthemia*. Moreover, DNA- and RNA-sequencing data revealed 19 RNA editing sites, including three synonymous and 16 nonsynonymous, in the chloroplast genome of *R. hybrida* that were distributed among 13 genes.

**Conclusions:**

The genome structure and gene content of *Rosa* chloroplast genomes are similar across various species. Phylogenetic analysis based on the *Rosa* chloroplast genomes has high resolution. Additionally, a total of 19 RNA editing sites were validated by RNA-Seq mapping in *R. hybrida*. The results provide valuable information for RNA editing and evolutionary studies of *Rosa* and a basis for further studies on genomic breeding of *Rosa* species.

**Supplementary Information:**

The online version contains supplementary material available at 10.1186/s12870-023-04338-0.

## Introduction

As a vital post-transcriptional regulation mechanism, RNA editing is pervasive in gene expression across chloroplast genomes of terrestrial plants [1, 2]. RNA editing typically involves conversion of cytidine (C) to uridine (U) within RNA molecules in the chloroplast genomes of higher plants [3, 4]. Reverse U-to-C editing has also been reported in plant organelle genomes, whereas U-to-C editing has been virtually absent in gymnosperms and angiosperms [1]. Most flowering plant chloroplast genomes have 20–60 RNA editing sites [5]. Chloroplast RNA editing sites decreased during angiosperm evolution [6–8]. Most RNA editing sites have been found in protein-coding regions, with a few sites located in untranslated regions, structural RNAs, and intronic regions [9]. Although the molecular mechanisms of RNA editing have been extensively studied [10], how RNA editing evolved in different species and about the mechanisms underlying the diversity of editing frequencies remain unclear. To date, relevant studies on detection of RNA editing sites via RNA-sequencing (RNA-Seq) read mapping and variant calling is lacking in the genus *Rosa*.

The genus *Rosa* L. (Rosaceae) contains approximately 200 species and grows in the subtropical and temperate regions of the northern hemisphere [11, 12]. Conventional taxonomy divided the genus *Rosa* into four subgenera (*Rosa*, *Hesperhodos*, *Hulthemia*, and *Platyrhodon*), while species of the subgenus *Rosa* are further divided into ten sections (*Rosa*, *Banksianae*, *Bracteatae*, *Caninae*, *Carolinae*, *Chinenses*, *Gallicanae*, *Pimpinellifoliae*, *Laevigatae*, and *Synstylae*) [13, 14]. *Rosa* species have extensive morphological variation and complex taxonomic profiles. In addition, reconstruction of the phylogeny of *Rosa* species has been difficult due to hybridization, incomplete lineage sorting, and low differentiation among the genus *Rosa* [15].

Chloroplasts are specialized plastids that contain chlorophyll to absorb light energy [16, 17]. Plant chloroplast genomes provide important information for exploring genetic diversity, understanding evolutionary differences, and generating high-resolution phylogenies, especially at low/complex taxonomic levels [18–20].

The chloroplast genome phylogenetic relationships of the genus *Rosa* still unclear because of the failure of species division, low resolution, limited samples, and low support values [15]. In the present study, the chloroplast genomes of three *Rosa* species, namely *R. hybrida* (Sect. *Chinenses*), *R. acicularis* (Sect. *Rosa*), and *R. rubiginosa* (Sect. *Caninae*), were assembled and compared. Among these three species, *R. acicularis* and *R. rubiginosa* have great medicinal importance, while *R. hybrida* is a commercial rose cultivar [21, 22]. Combined with the previously reported 41 chloroplast genomes of *Rosa*, we performed a comprehensive chloroplast genome analysis of this taxonomically difficult plant taxon. Furthermore, to our knowledge, we have for the first time determined RNA editing sites in the whole chloroplast genome of *R. hybrida* (commercial rose cultivar) using RNA-Seq data. This study aimed to (1) perform a comparative analysis of the chloroplast genomes of *Rosa* species; (2) ascertain highly variable regions in the *Rosa* chloroplast genome sequences; (3) identify chloroplast gene insertion in mitochondria; (4) obtain early evolutionary information on the chloroplast genomes of Rosa species and analyze molecular phylogeny by comparing chloroplast genomes; and (5) identify RNA editing sites of *R. hybrida* using RNA-Seq data. This study will provide a better understanding of the interspecific differences in the genus *Rosa* and will be valuable for further research on RNA editing in *Rosa* species.

## Results

### Characteristics of the *Rosa* chloroplast genomes

**Fig. 1:**
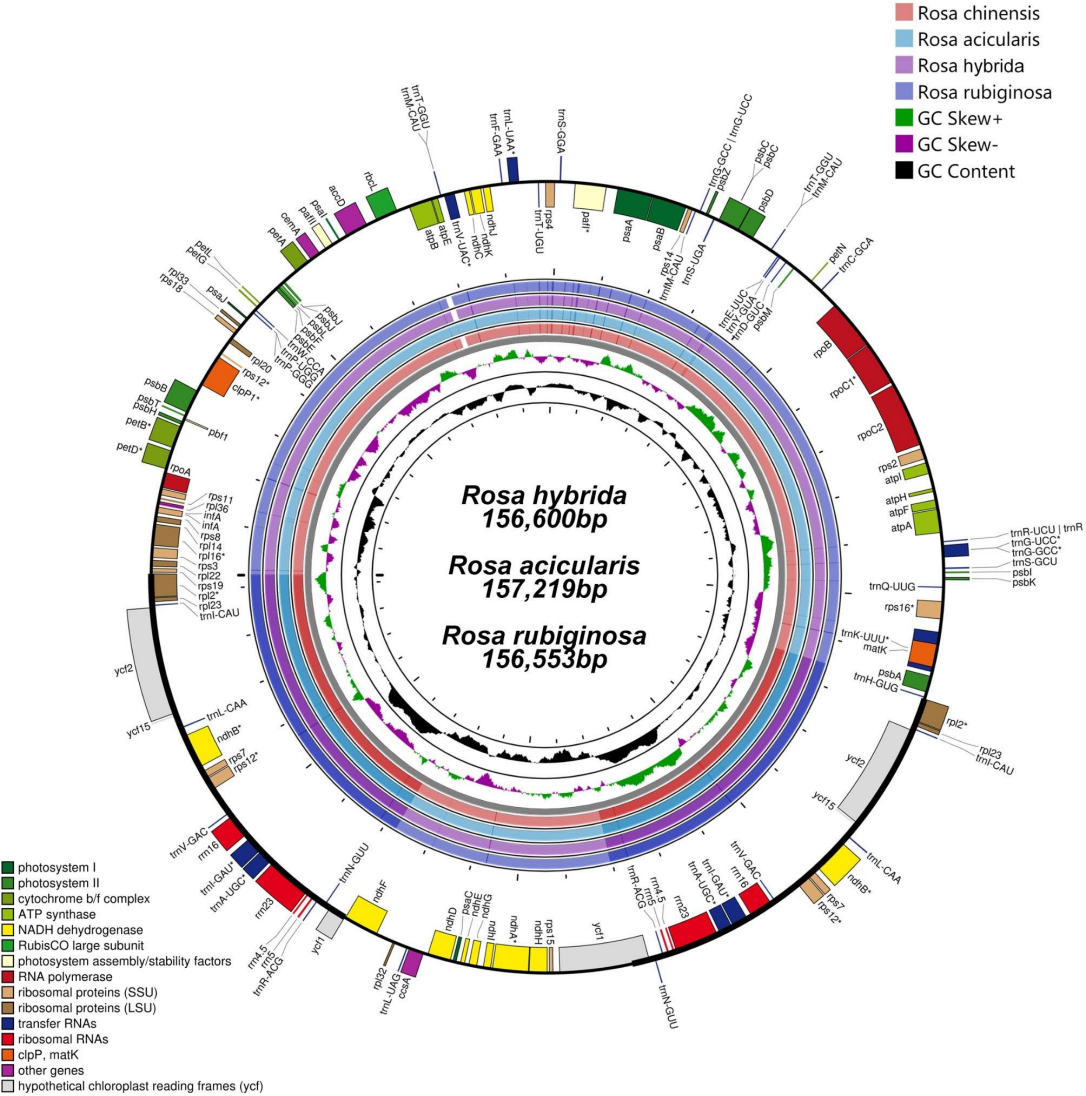
Map of aligned *Rosa* chloroplast genomes. Gene map of the *Rosa* chloroplast genomes, sequence alignment of *Rosa* species chloroplast genomes with *R. rugosa* as the reference, GC skew, and GC content from outside to inside. The circular map was drawn using OGDraw

**Fig. 2:**
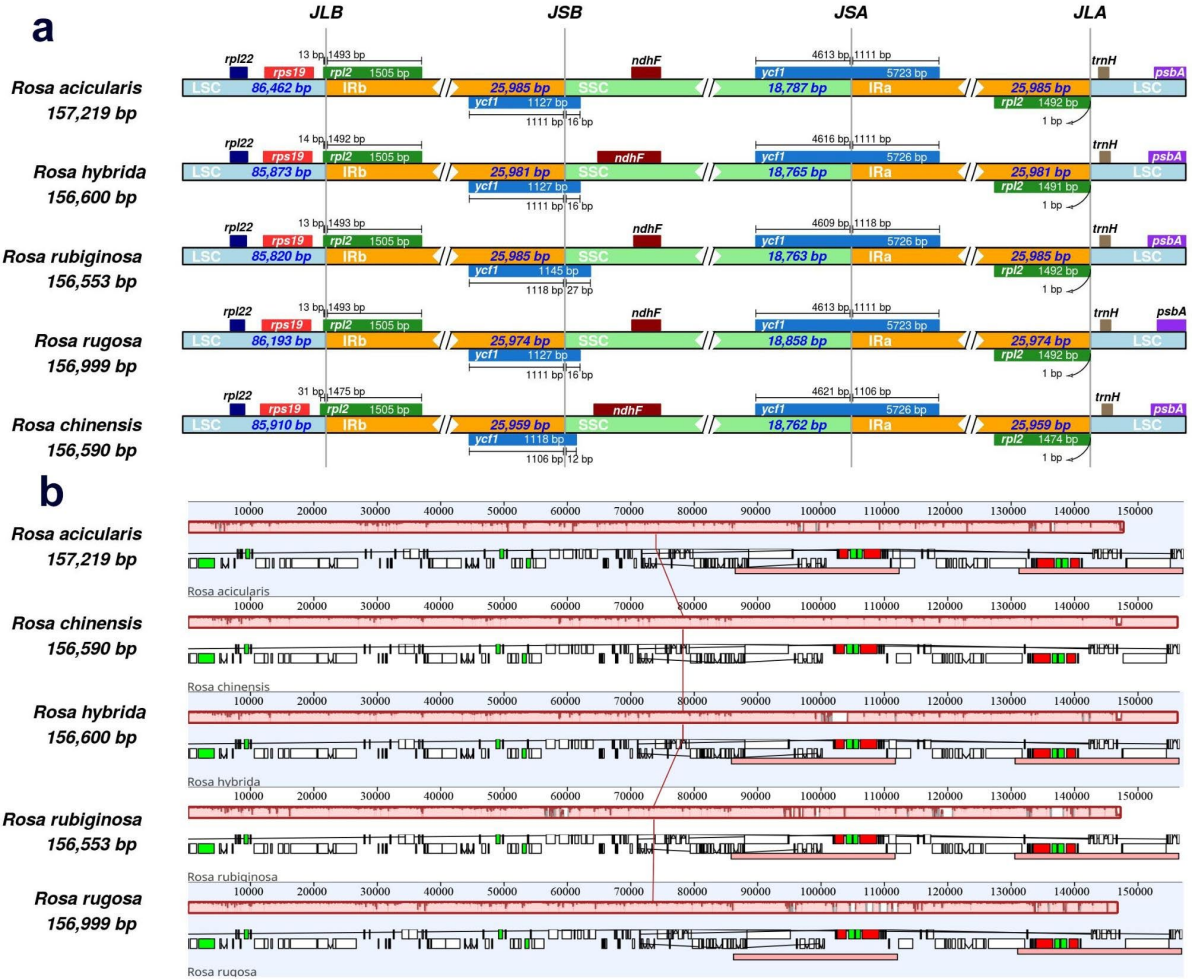
Comparative analysis of five *Rosa* chloroplast genomes. (**a**) Comparison of the borders of large single-copy, inverted repeat, and small single-copy regions among the five *Rosa* genomes. Colored boxes indicate the genes across the junctions. (**b**) *Rosa* chloroplast genome collinearity comparison plot. Local co-linear blocks (LCB) were colored to indicate regions of commonality. The histogram within each block indicates the degree of sequence similarity. The results were visualized by IRscope and Mauve

Raw sequence data of *R. rubiginosa*, *R. hybrida*, and *R. acicularis* were obtained, and the chloroplast genomes were 156,553 bp, 156,600 bp, and 157,219 bp long, respectively (Fig. 1). The three newly assembled *Rosa* chloroplast genomes were deposited in the GenBank database (OP032236, OP032237, and OP032238). They exhibited a quadripartite structure with a large single-copy (LSC) region (85,820–86,462 bp), dual inverted repeat (IR) regions (25,981–25,985 bp), and a small single-copy (SSC) region (18,763–18,787 bp), as shown in Fig. 2a.

The chloroplast genomes of *R. rubiginosa*, *R. hybrida*, and *R. acicularis* were conserved and contained 115 unique genes, of which 80 were protein-coding genes, 31 were transfer RNA (tRNA) genes, and 4 were ribosomal RNA (rRNA) genes. Seventeen genes had introns, of which eight protein-coding genes (*rpl16, rpl2, rps16, rpoC1, petB, petD, ndhA*, and *ndhB*) and six tRNAs (*trnG-UCC, trnI-GAU, trnK-UUU, trnL-UAA, trnA-UGC*, and *trnV-UAC*) contained one intron while the other three genes (*pafI, clpP1*, and *rps12*) had two introns.

### Comparative analysis of *Rosa* chloroplast genomes

**Fig. 3:**
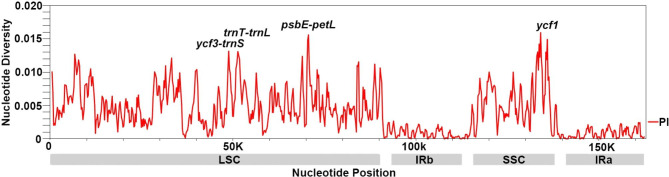
Sliding window analysis of the *Rosa* chloroplast genomes using the DnaSP program. Window length: 600 bp; step size: 200 bp. X-axis, Position of a window; Y-axis, Nucleotide diversity per window

**Fig. 4:**
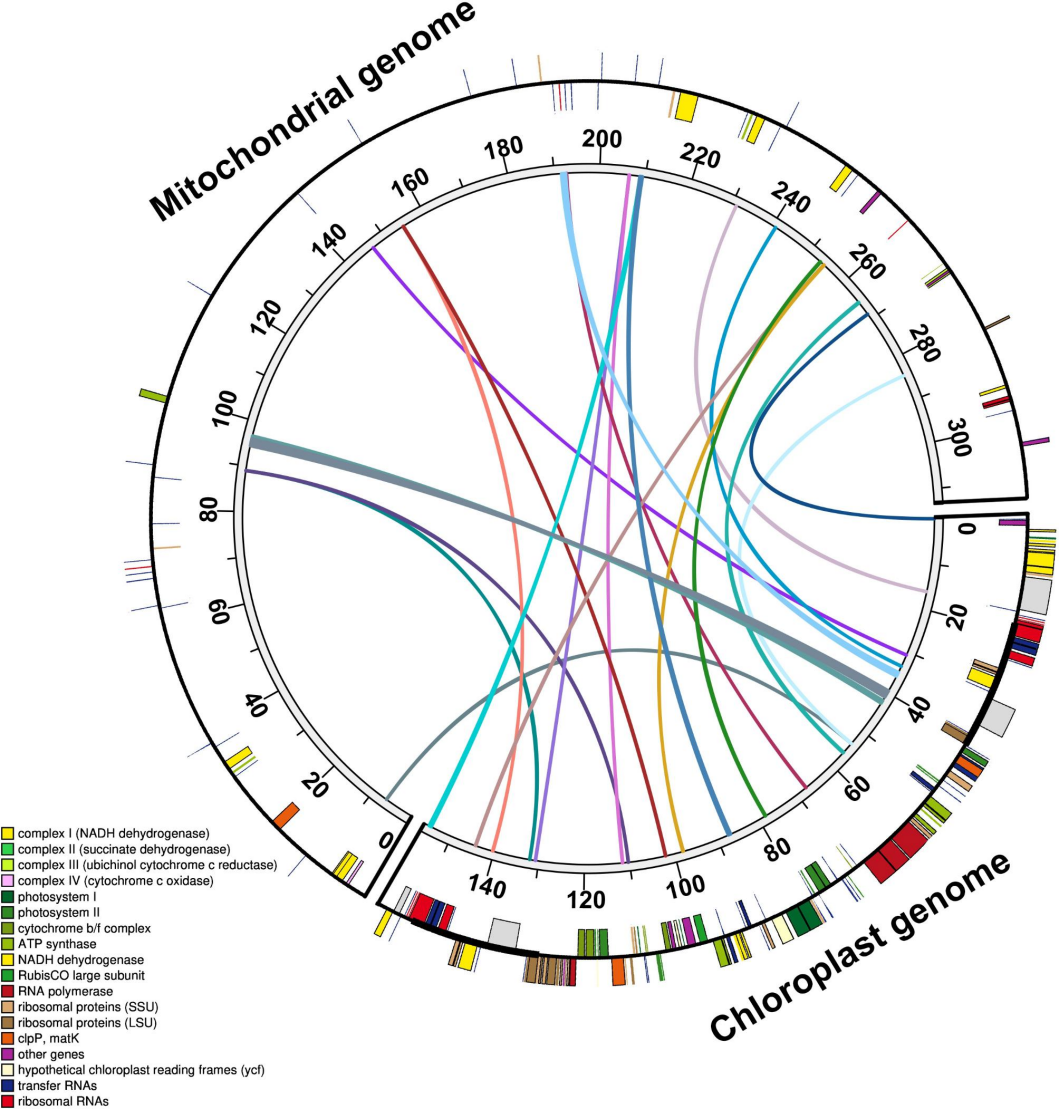
Schematic diagram of gene transfer between mitochondrial and chloroplast genomes in *R. chinensis*. Colored lines within the circle show where the chloroplast genome segment entering the mitochondrial genome. Genes within a circle are transcribed clockwise, while those outside the circle are transcribed counterclockwise. The gene transfer results were visualized using Circos

The *Rosa* chloroplast genomes had high sequence similarity. By comparison of the expansion and contraction of the IR/SC boundary between the chloroplast genomes of *Rosa*, it can be seen that the *Rosa* chloroplast genomes shows high similarity at the IR/SC boundary (Fig. 2a). The *rpl2* gene contained all LSC/IRb junctions, and the boundary gene between SSC and IRa/IRb is *ycf1*. Overall, the *Rosa* IR regions are similar in length and structure, which is consistent with previous findings [23, 24]. Similar to most terrestrial plants, the IR regions of chloroplast genome were more conserved than the LSC and SSC regions, and noncoding regions exhibited relatively higher sequence differentiation than gene-coding regions (Figs. 1 and 3) [25]. Additionally, there were no gene rearrangements, inversions, or losses among the chloroplast genomes of the five *Rosa* species (Fig. 2b). There were some highly variable regions in the chloroplast genome sequences that were often clustered together and were referred to as “hotspots” [26]. Next, nucleotide substitution and nucleotide diversity (Pi) values for 24 *Rosa* chloroplast genomes (Table S1) were calculated to identify sequence divergence hotspots (Figs. 3 and 4). A nucleotide substitution search of 24 *Rosa* chloroplast genomes identified 3,173 (1.95%) variable sites, including 1,426 (0.88%) parsimony-informative sites. The Pi values were in the range of 0–0.016, with high values (Pi > 0.013) in the following regions: *ycf3-trnS, trnT-trnL, psbE-petL*, and *ycf1*. The hotspot regions could be used as molecular markers for differentiation in *Rosa* species.

### Gene transfer between the chloroplast and mitochondrial genomes

The length of the mitochondrial genome sequence for *R. chinensis* in GenBank was found to be approximately twice as large as the chloroplast genome. Additionally, 22 chloroplast genomic fragments with a total length of 6,192 bp and > 90% sequence similarity with their counterparts were identified in the mitochondrial genome, representing 3.96% of the chloroplast genome (Fig. 4 and Table S2). Two complete mitochondrial protein-coding genes (*psbC* and *rpl23*) and four tRNAs genes (*trnW-CCA, trnN-GUU, trnH-GUG*, and *trnM-CAU*) were identified.

### Phylogenetic relationship based on chloroplast genomes

**Fig. 5:**
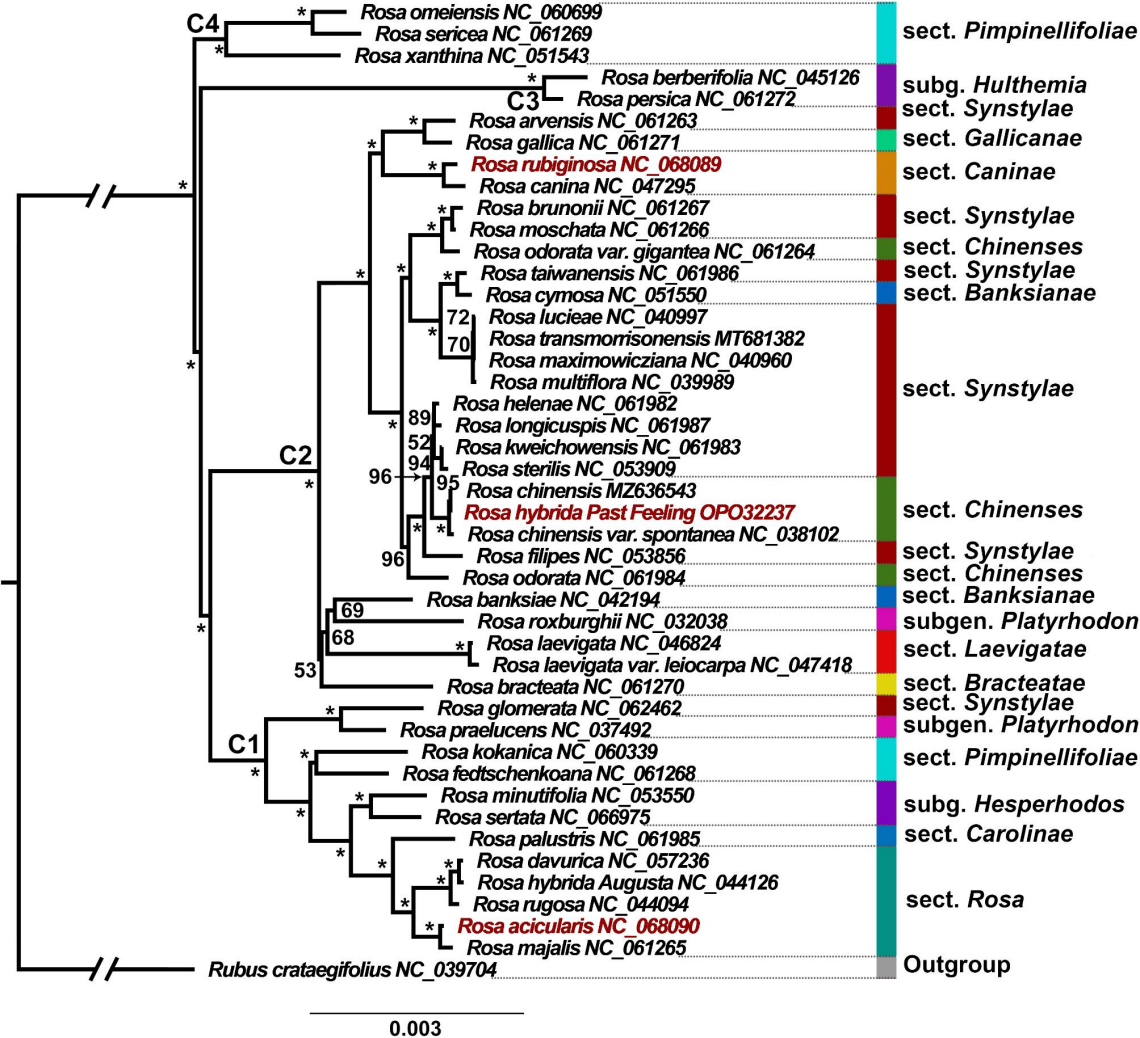
Maximum Likelihood (ML) phylogenetic tree reconstruction of 44 *Rosa* species based on whole chloroplast genome sequences using IQ-TREE. The best-fit substitution model (TVM + F + I + G4) was used to build phylogenetic tree. Bootstrap resampling with 1,000 replicates was employed to assess branching support. Numbers with branches indicate ML bootstrap values, asterisk denotes 100% ML bootstrap support. *Rubus crataegifolius* was used as the outgroup. The GenBank numbers of all species are shown in the figure. Different colors correspond to the section names

The chloroplast genomes of the 44 *Rosa* species were used to infer their phylogenetic location, except for the three newly assembled chloroplast genomes, the complete chloroplast genome sequences of 41 *Rosa* species were obtained from the National Center for Biotechnology Information (NCBI) database. Most Maximum Likelihood (ML) tree nodes had bootstrap support values of 100% (Fig. 5). Four well-supported clades (C1, C2, C3, and C4 Clade) were recovered within *Rosa*. C1 Clade included sections *Rosa*, *Carolinae*, *Hesperhodos*, and two species from section *Pimpinellifoliae, Rosa glomerata* (Sect. *Synstylae*) and *Rosa praelucens* (Subg. *Platyrhodon*) were nested in C1 Clade. C2 Clade included most samples from section *Synstylae*, all samples from sections *Bracteatae, Laevigatae, Banksianae, Chinenses, Caninae, Gallicanae*, as well as one species from subgenus *Platyrhodon* (*R. roxburghii*). The *Hulthemia* species formed C3 Clade. C4 Clade includes three species from section *Pimpinellifoliae* (*R. omeiensis*, *R. sericea*, and *R. xanthina*).

### Identification of RNA editing sites using RNA-Seq data

**Fig. 6:**
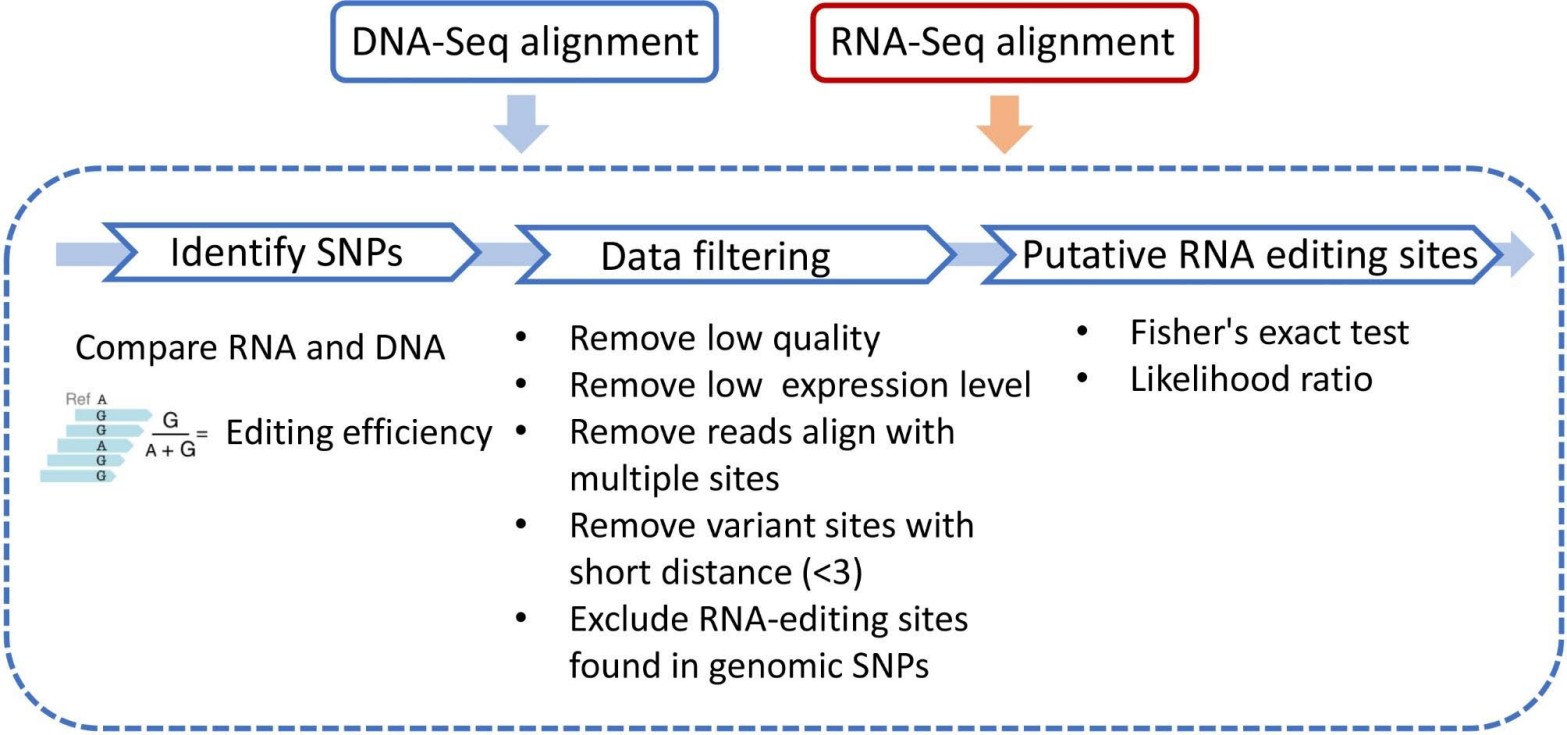
Overview of the RNA editing site identification and analysis pipeline

RNA editing sites in *R. hybrida* ‘Past Feeling’ were identified via RNA-Seq data mapping. A 99% region of the organellar transcripts were covered by reads, and the average sequencing depth was over 52x. In addition, the distribution of reads was uneven. The genome coverage maps are shown in Figure S1. Using a stringent screening procedure described in Materials and Methods (Fig. 6), we identified a total of 19 RNA editing sites in the chloroplast genome (Table 1). All of the editing sites were C-to-U conversions and were located in protein-coding regions. The 19 RNA editing sites in the chloroplast genome were distributed among 13 genes and included three synonymous and 16 nonsynonymous RNA editing sites. Most RNA editing sites occurred at the second codon position. RNA editing at the first and second codon positions resulted in amino acid conversion, whereas that at the third codon position resulted in silent changes, e.g. proline (CCC) to proline (CCU). However, silent codon changes only accounted for 15.79% of the total number of RNA editing sites in the chloroplast genome. The RNA editing efficiency ranged from 38.89 to 100% with a mean of 82.96%. Compared with the RNA editing of the *Arabidopsis* chloroplast genome [27], six conserved RNA editing sites (*rps14*-27, *rps14*-50, *accD*-264, *clpP1*-187, *rpoA*-277, and *ndhD*-128) were identified in the *R. hybrida* chloroplast genome, accounting for 31.58% of the total number of RNA editing sites.

**Table 1:** RNA editing sites in the chloroplast genome of *R. hybrida* identified using RNA-sequencing data

Gene name	Editing position in genome	Editing position in gene	Editing position in codon	Editing type	Codon Change	Amino Acid Change	Coverage Depth	Editing efficiency
*atpA*	10,866	1263	3	C->U	CUC->CUU	L->L	9	88.89%
*rps14*	37,798	80	2	C->U	UCA->UUA	S->L	50	92.00%
*rps14*	37,729	149	2	C->U	CCA->CUA	P->L	37	97.30%
*accD*	59,505	791	2	C->U	UCG->UUG	S->L	18	38.89%
*psaI*	60,728	83	2	C->U	UCU->UUU	S->F	13	84.62%
*psaI*	60,733	88	1	C->U	CAU->UAU	H->Y	13	69.23%
*petL*	67,058	5	2	C->U	CCU->CUU	P->L	42	80.95%
*rps18*	69,519	221	2	C->U	CCG->CUG	P->L	27	74.07%
*clpP1*	71,381	559	1	C->U	CAU->UAU	H->Y	14	100.00%
*psbB*	74,290	414	3	C->U	AUC->AUU	I->I	117	69.23%
*petB*	77,760	611	2	C->U	CCA->CUA	P->L	53	73.58%
*rpoA*	80,192	200	2	C->U	UCU->UUU	S->F	9	77.78%
*rpoA*	79,562	830	2	C->U	UCA->UUA	S->L	14	71.43%
*rpl16*	83,113	12	3	C->U	CCC->CCU	P->P	24	75.00%
*ndhD*	118,698	383	2	C->U	CCA->CUA	P->L	5	100.00%
*ndhA*	121,876	961	1	C->U	CCU->UCU	P->S	10	100.00%
*ndhA*	121,764	1073	2	C->U	UCC->UUC	S->F	12	91.67%
*ndhA*	121,876	2053	1	C->U	CCU->UCU	P->S	10	100.00%
*ndhA*	121,764	2165	2	C->U	UCC->UUC	S->F	12	91.67%

## Discussion

The chloroplast genomes of the *Rosa* species were generally consistent in terms of genomic structure, gene number, type, and order, with the exception of some single nucleotide polymorphisms (SNPs) and insertion and deletion variations [15, 23, 28, 29]. There is gene loss in the evolution of plant chloroplast genome [30], while there is a high level of conservation in the genus *Rosa* suggests evolutionary constraint in the chloroplast genome, which is prevalent in higher plants [25].

Unlike the nuclear genome, the chloroplast genome has multiple copies in the cell and is smaller in size. In addition, chloroplast genomes have sufficient interspecific differentiation. Therefore, the use of chloroplast genome sequences is one of the best approaches for species identification at present [31]. In this study, based on the results of the alignment of *Rosa* chloroplast genomes and SNP analysis, we found an increased number of variable sites in the four specific regions, namely *ycf3-trnS, trnT-trnL, psbE-petL*, and *ycf1*. Thus, using these regions as novel candidate segments may provide useful information for *Rosa* species identification. However, further experiments are needed to support these results.

Intracellular gene transfer occurs between the nucleus, mitochondria, and chloroplast [32, 33]. Gene transfer among mitochondrial and chloroplast genomes is common during the long-term evolution of plants [32, 34]. Intracellular gene transfer may be responsible for the high rearrangement of the mitochondrial genome, because the chloroplast genome segment entering the mitochondria was highly aligned with the original chloroplast genome sequences and the insertion position of the segments were randomly located [35]. The total length of these transferred fragments in Rosa mitochondrial genome was 6,192 bp, this is much shorter than the transfer fragments we found in other genera [36], this may be one of the reasons why the mitochondrial genome of Rosa is relatively small.

In this study, a phylogenetic tree based on chloroplast genome sequences was constructed to explore the evolutionary relationship in the genus *Rosa* and was found to be generally consistent with previously reported results [13, 29, 37]. There were several inconsistencies between the nuclear and chloroplast phylogenetic topology, particularly the position of section *Rosa*, which may be due to incomplete lineage, differences in the evolutionary rates of chloroplast and nuclear genes, or introgressive hybridization [37]. The earliest divergence in the chloroplast phylogeny roughly distinguished species of sections *Pimpinellifoliae* and *Rosa* and subgenera *Hulthemia*, *Platyrhodon*, and *Hesperhodos* from species of sections *Synstyale*, *Laevigatae*, *Banksianae*, *Caninae*, and *Chinenses*, which is consistent with previous studies [37, 38].

RNA editing of the *Rosa* chloroplast genome is one of the focal points of this study. As a vital post-transcriptional regulation mechanism, it has been generally accepted that 20–60 RNA editing sites are present in most chloroplast genomes [1, 39]. Previously, a software was used to predict RNA editing sites; however, its accuracy rate was generally low, and synonymous mutation sites could not be predicted. The advent of next-generation sequencing (NGS) has improved the sensitivity and accuracy of RNA editing site identification [40, 41]. In this study, similar to many plant organellar genome RNA editing studies [41–43], the data was obtained through the polyA RNA protocol. Since plant organellar transcripts generally do not have poly-A tail [44], the editing efficiency can be biased. Nonetheless, RNA-seq data obtained by polyA RNA protocol have implications in RNA editing studies of organelle genome [44]. In the present study, all editing sites found were C-to-U conversions. Furthermore, no editing sites were observed in tRNA and rRNA genes. These may be due to the stringent filtering process in our identification pipeline. Each species has its own unique RNA editing sites in comparison with other species, which indicates that RNA editing sites are independently lost after species divergence. Overall, the codon preference of targets for RNA editing, the tendency of increased protein hydrophobicity, and site distribution showed similar trends across species.

## Conclusions

In conclusion, we assembled and compared the chloroplast genomes of *Rosa* species and found that the genome structure and gene content of *Rosa* chloroplast genomes are similar across various species. We also identified 22 chloroplast fragments in the mitochondrial genome. Phylogenetic analysis based on the *Rosa* chloroplast genomes has high resolution. Additionally, a total of 19 RNA editing sites in 13 genes were validated by RNA-Seq mapping in *R. hybrida*. The findings of this study provide valuable genetic resources for further research on *Rosa* species.

## Materials and methods

### Plant material and sequencing

The *Rosa* accessions were from the *Rosa* nuclear genome and transcriptome sequencing projects (Table S3). Total genomic DNA was extracted from herbarium (*R. acicularis, R. rubiginosa*) or petals (*R. hybrida*) using the CTAB method. The voucher specimens of *R. acicularis* (TROM_V_91069) and *R. rubiginosa* (TROM_V_148853) and leaves were used for DNA extraction. Petals were provided by Kunming Yangyueji Company. Paired − end (2 × 100 bp) genomic libraries were constructed using Illumina kit for sequencing on BGISEQ − 500 and Illumina hiseq 2500 sequencers with an average insertion size of 300 bp. Total RNA was extracted from petals using the SV total RNA Isolation Kit (Promega, WI, USA). The method of rRNA depletion is poly-A selection, which relies on the use of Oligo (dT)-attached magnetic beads to isolate protein-coding polyadenylated RNA transcripts. A NEBNext® UltraTM RNA Library Prep Kit (New England Biolabs, MA, USA) was used to generate libraries and sequenced on an Illumina HiSeqTM 2000 instrument at Novogene Bioinformatics Technology Co., Ltd. (Beijing, China). The raw chloroplast genomes and transcriptome sequencing data were uploaded in the NCBI sequence read archive with accession numbers SRR21561260–SRR21561263.

### Chloroplast genome assembly and annotation

Raw sequencing data were filtered using Trimmomatic v0.38 [45]. *De novo* assembly was then performed using SPAdes version 3.61 with different k-mer parameters [46]. Next, the Geneious Prime software v2022.2 [47] was used to order *de novo* scaffolds that were positively correlated with chloroplasts on to the reference chloroplast genome of *R. rugosa* (NC_044094).

GeSeq was used to perform chloroplast genome annotation to predict gene-coding proteins, rRNAs, and tRNAs, with manual curation as needed [48]. Subsequently, the circular map of the *Rosa* chloroplast genome was drawn using OGDraw v1.3.1 [49].

### Genome comparative analysis and hotspots regions screening

*Rosa* chloroplast genome sequences were aligned using MAFFT v7.221 [50]. Comparison of the borders of LSC, IR and SSC regions among the five *Rosa* genomes (OP032236, OP032237, OP032238, MK986659, and NC_038102) was visualized by IRscope [51]. The Mauve multiple genome alignment method was used to detect rearrangements and co-linearities in the chloroplast genomes of the five *Rosa* species [52]. To examine the rapidly evolving molecular markers among *Rosa* species, we used 24 *Rosa* chloroplast genomes (Table S1) for the sliding window analysis with a window size of 600 bp and a step length of 200 bp using DnaSP v6.12 [53].

### Identification of chloroplast gene insertion in mitochondria

The mitochondrial and chloroplast genomes of *R. chinensis* were retrieved from GenBank (CM009589 and CM009590, respectively). The genes transferred between the mitochondrial and chloroplast genomes were then identified via homology searches using Basic Local Alignment Search Tool. Chloroplast and mitochondrial maps of *Rosa* and fragments of gene transfer were visualized using Circos [54].

### Phylogenetic analysis

Phylogenetic trees were constructed using the whole chloroplast genome sequences of 44 *Rosa* species to identify their genetic relationship. *Rubus crataegifolius* was used as the outgroup. Genome sequences were aligned using MAFFT v7.221 [50], and all alignments were manually inspected and adjusted. IQ-TREE v 1.6.12 [55]was used to build an ML phylogenetic tree with the best-fit substitution model (TVM + F + I + G4) determined by ModelFinder v3.7 [56]. Bootstrap resampling with 1,000 replicates was employed to assess branching support.

### Identification of RNA editing sites using RNA-Seq data

The clean RNA-Seq reads were aligned to the chloroplast genome of *R. hybrida* ‘Past Feeling’ using the Hisat2 v2.1.0 tool [57]. To convert sequence alignment map to binary alignment map, the samtools v1.9 view command was used [58]. Potential RNA editing sites were extracted using the SNP calling method in bcftools v1.9 [58]. Extracted SNPs were then processed with REDO v1.0 to provide annotation information for editing sites [59]. To eliminate the false positive RNA editing sites, DNA-Seq reads of *R. hybrida* ‘Past Feeling’ were aligned to the chloroplast genome using Bowtie 2 v2.3.5 [60]. Genomic SNP-calling was performed using bcftools v1.9 [58]. RNA editing sites that were found in genomic SNPs were then excluded (Fig. 6).

## Electronic supplementary material

Below is the link to the electronic supplementary material.

Supplementary Material 1

Supplementary Material 2

## Data Availability

The data supporting the findings of this study are freely available in GenBank on the NCBI website at https://www.ncbi.nlm.nih.gov, using the accession number OP032236, OP032237, and OP032238. Raw sequencing data have been deposited at the NCBI Sequence Read Archive (SRA) under accession SRR21561260–SRR21561263.

## Abbreviations

RNA-Seq: RNA-sequencing
SSC: Small single copy
LSC: Large single copy
IR: Inverted repeat
tRNA: Transfer RNA
rRNA: Ribosomal RNA
ML: Maximum Likelihood
SNPs: Single nucleotide polymorphisms
NGS: Next-generation sequencing
