# Supplementary figures and images for: Comparative analysis of the chloroplast genomes of Rosa species and RNA editing analysis
Source: BMC Plant Biol. 2023 Jun 14;23:318. doi: 10.1186/s12870-023-04338-0 (PMC10265813; doi:10.1186/s12870-023-04338-0)

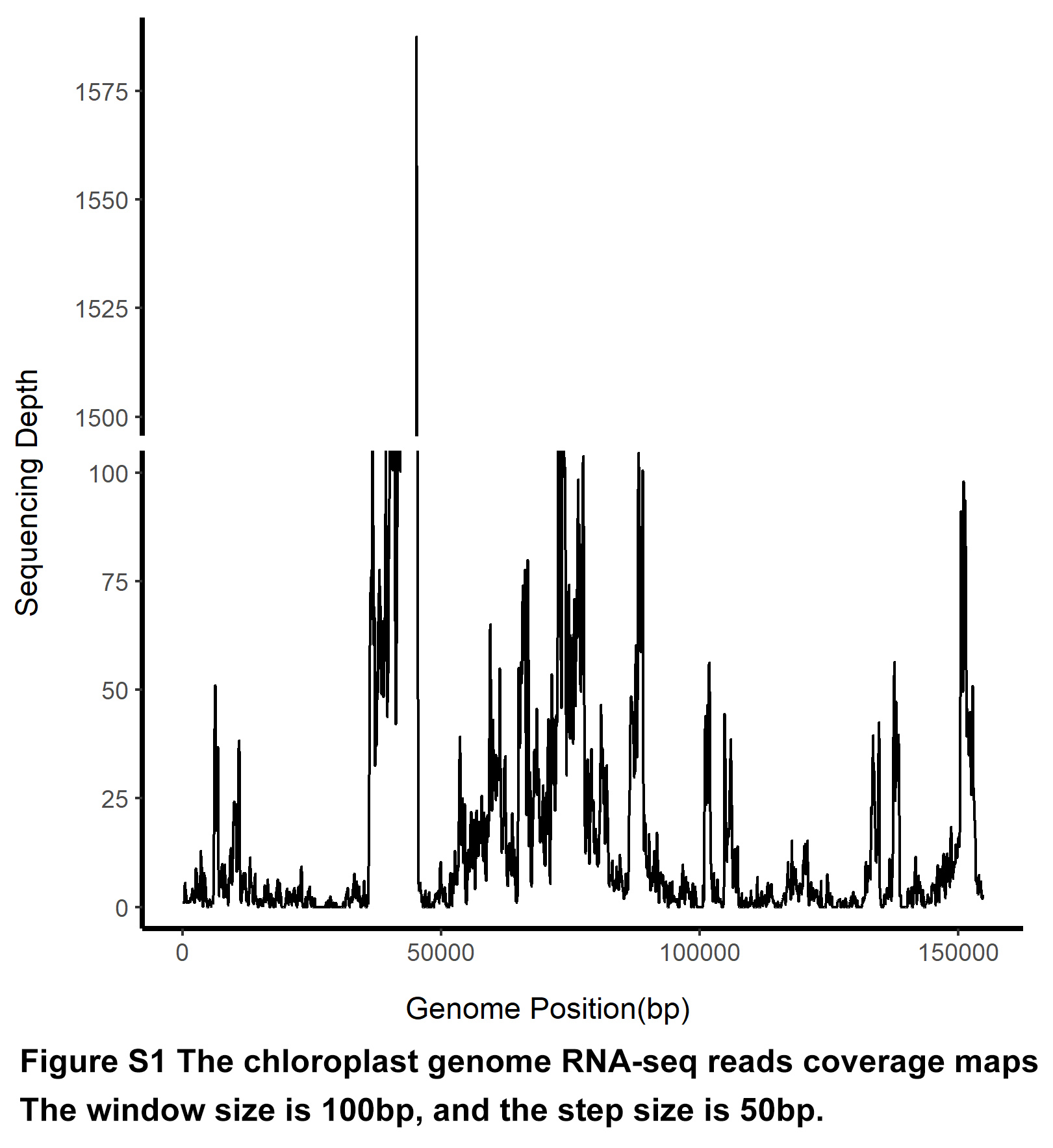

Supplement: Supplementary file 1 — Supplementary Material 1 [file 12870_2023_4338_MOESM1_ESM.jpg]
